# The London General Hospitals and the Central Hospital Board

**Published:** 1897-06-12

**Authors:** 


### THE LONDON GENERAL HOSPITALS AND THE CENTRAL HOSPITAL BOARD.

It is an open secret that several meetings have recently taken place of the representatives of the hospitals of London with medical schools attached, including those of St. Bartholomew's, St. Thomas's, Guy's, Charing Cross, King's College, the London, Middlesex, Royal Free, St. George's, St. Mary's, University College, and Westminster Hospitals, to consider the action that the large general hospitals should take on the question of the proposal to establish a Central Hospital Board for London. We understand that this conference of representatives of the hospitals of London with medical schools attached, duly authorised to express the opinion of their respective boards or committees of management, have unanimously resolved to deprecate the endeavour now being made to connect the existence of the Prince of Wales's Fund with the proposal to form a Central Hospital Board for London on the lines that have been made public, and to adminster the funds. They further unanimously determined to deprecate the establishment of a Central Hospital Board having any control over the administration of the finances of the hospitals or medical schools of the metropolis. The conference further determined to take suitable measures for acquainting H.R.H. the Prince of Wales with these resolutions, and at the same time to assure H.R.H. of the profound gratitude of the hospitals for his kind and gracious efforts on their behalf.

